# Betaine, a component of *Lycium chinense*, enhances muscular endurance of mice and myogenesis of myoblasts

**DOI:** 10.1002/fsn3.2466

**Published:** 2021-07-14

**Authors:** Sang‐Soo Lee, Yong‐An Kim, Bokkee Eun, Jayeon Yoo, Eun‐Mi Kim, Myoung Soo Nam, Kee K. Kim

**Affiliations:** ^1^ Department of Biochemistry Chungnam National University Daejeon Korea; ^2^ Core Laboratory for Convergent Translational Research Korea University College of Medicine Seoul Korea; ^3^ National Institute of Animal Science RDA Wanjugun Korea; ^4^ Department of Predictive Toxicology Korea Institute of Toxicology Daejeon South Korea; ^5^ Division of Animal Resource Science Chungnam National University Daejeon Korea

**Keywords:** cheese, gastrocnemius muscle, *Lycium chinense*, myogenesis, sarcopenia

## Abstract

Sarcopenia is a disease characterized by the loss of muscle mass and function that occurs mainly in older adults. The present study was designed to investigate the hypothesis that water extract of *Lycium chinense* (WELC) would improve muscle function and promote myogenesis for sarcopenia. We investigated the effect of water extracts of *L*. *chinense* on muscular endurance function and myogenesis to examine its efficacy in sarcopenia. Intake of WELC‐containing cheese enhanced the muscular endurance function of mice in treadmill endurance tests. In addition, the cross‐sectional areas of muscle fibers in the gastrocnemius muscle of *L*. *chinense*‐fed mice were greater than that of control mice. Furthermore, WELC and its key component marker substance betaine promoted myogenesis of myoblasts by increasing the expression of the myogenic protein myosin heavy chain 3 (Myh3) and myotube formation. Taken together, our results suggest that *L*. *chinense* may potentially be useful in the development of preventive and therapeutic agents for sarcopenia, as well as in providing basic knowledge on myogenesis and muscular functions.

## Graphical abstract

WELC and its key component marker substance betaine promoted myogenesis of myoblasts by increasing the expression of the myogenic protein myosin heavy chain 3 (Myh3) and myotube formation. *L*. *chinense* may potentially be useful in the development of preventive and therapeutic agents for sarcopenia, as well as in providing basic knowledge on myogenesis and muscular functions.

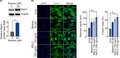

## 1 INTRODUCTION

Muscle formation occurs during development, and during regeneration of muscle tissue following damage by exercise or disease (Menko & Boettiger, 1987; Yu et al., 2014).

During the process of muscle regeneration, myocardial cells exit the stationary/quiescence state and differentiate to form myofibers through cell fusion. This differentiation process is called myogenesis and is controlled by various signaling cascades. Various myogenic proteins, such as paired Box Protein Pax‐7 (Pax7), myoblast determination protein 1 (MyoD), myogenin (MyoG), and myosin heavy chain 3 (Myh3) represent the different myogenic stages (Rudnicki et al., 1992; Weintraub et al., 1989; Wright et al., 1989). Myoblasts form thick and long myotube cells through the process of myogenesis and the expression of myogenic proteins and the length and thickness of myotubes are very important factors in assessing myogenesis. Suppression of myogenesis due to various factors, such as aging, inhibits myotube formation that prevents the normal formation of muscle tissues, including skeletal muscle, resulting in muscle loss that leads to reduction in the total amount of muscles (Bodine et al., 2001; Janssen et al., 2004; Suetta et al., 2013).

Muscle loss is a typical muscle and nervous system disease and is one of the symptoms of aging. The total amount of skeletal muscle decreases gradually due to the natural process of aging and is replaced by adipose tissue resulting in a condition called sarcopenia (Roche, 1994). Sarcopenia mainly occurs in adults over the age of 65 years, and the number of older adults is increasing (Bae & Kim, 2017; von Haehling et al., 2010). Sarcopenia decreases the total amount of skeletal muscles and consequently increases the risk of fractures due to insufficient protection of the bones by skeletal muscles leading to decrease in the amount of activity and metabolic diseases such as diabetes, and thus has a tremendous impact on human health (Butcher et al., 2018; Leslie et al., 2020). Although sarcopenia is influenced by several factors, such as genetics or drugs, it is also affected by diet. Recent studies have confirmed that muscle function is improved by eating cheese with high protein and saturated fatty acid content (Alemán‐Mateo et al., 2012; Devries & Phillips, 2015). In addition, the effect of various natural products on myogenesis are being actively investigated (Chen et al., 2019; Cho et al., 2020; Lee et al., 2020).

*Lycium chinense* is a medicinal herb cultivated throughout Asia. It protects the liver, has beneficial effects in osteoporosis, and has been used as a herbal medicine for its anticancer and antioxidant properties (Mocan et al., 2014). The efficacy of *L*. *chinense* is due to its high content of polyphenols, carotenoids, and amino acids (Kruczek et al., 2020; Thiruvengadam et al., 2020). *L*. *chinense* also contains betaine, a natural amino acid and a precursor of methionine. Although betaine has been studied for its role in improving muscle function, the effect of *L*. *chinense* with a high content of betaine on myogenesis and muscle function has not yet been studied (Cholewa et al., 2018; Senesi et al., 2013).

In this study, the effect of water extract of *L*. *chinense* (WELC) on muscle endurance and muscle formation was investigated. In addition, treatment with WELC and betaine was shown to influence myogenesis in C2C12 and primary myoblasts.

## 2 MATERIALS AND METHODS

### 2.1 Animals and experimental diets

BALB/c mice (8‐week‐old male; Daehan Biolink) were housed at 22 ± 2 ℃, with 50% ± 5% relative humidity under 12 hr light/dark cycle with ad libitum access to standard chow diet and water. All mice were acclimated for 7 days before use in experiments. Mice were divided into three groups (*n* = 4 mice/group) as follows: standard chow diet (Cont.), standard chow diet containing 40% cheese (Che.), and Che. with 3% *L*. *chinense* (Che. + LC). During the 6‐week feeding period, body weight was recorded every 7 days, and food intake was recorded every 3 days.

### 2.2 Extracts, asiago cheese, experimental diets, and chemical preparation

*Lycium chinense* was gifted by the Gogi Berry Research Institute, Cheongyang, Republic of Korea. Dried *L*. *chinense* (25 g) was extracted with distilled water (1 L) at boiling temperature for 2 hr. After the water evaporated, the extracts were freeze‐dried. Six month‐fermented asiago cheese, and 6 month‐fermented asiago cheese containing 3% *L*. *chinense* extract were used. Betaine (Sigma Aldrich) was used as a marker for *L*. *chinense*. The Asiago Cheese was prepared by the Segato et al. method (Segato et al., 2007). The amount of added *L*. *chinense* extract was 0% and 3.0%. Ripening of the control of Asiago Cheese was performed for 6 months in the ripening room (10 ± 2 °C and 85 ± 5% of relative humidity).

### 2.3 Exercise treadmill test

The exercise treadmill test was performed at a rate of 10 cm/s for 3 min with a 0% slope. The speed was gradually increased to 15 cm/s and then maintained at 45 cm/s until the mouse was unable to run for more than 10 s.

### 2.4 Serum biochemical analysis

The effects of the experimental diets on serum glutamic‐oxaloacetic transaminase (GOT), glucose, blood urea nitrogen (BUN), total cholesterol, triglyceride, high‐density lipoprotein‐cholesterol (HDL‐C), and low‐density lipoprotein‐cholesterol (LDL‐C) were measured using an auto‐analyzer (TBA‐40FR; Toshiba).

### 2.5 Cell culture

C2C12 cells were cultured in Dulbecco's modified Eagle's medium supplemented with 10% heat‐inactivated fetal bovine serum (Gibco) and 1% streptomycin/penicillin at 37 °C in a humidified 5% CO_2_ atmosphere. When the cells reached 90% confluence, the culture medium was replaced with Dulbecco's modified Eagle's medium supplemented with 2% horse serum (Gibco) and 1% streptomycin/penicillin. While inducing myogenesis, the medium was replaced with fresh medium every day.

### 2.6 PLO and collagen coating

To coat the myoblast culture dishes or plates with poly‐L‐ornithine (PLO, Sigma) and collagen (Santa Cruz Biotechnology), the dishes and plates were incubated 6 hr at 25 ℃ with 0.001% solution of PLO in sterile water at 5 µg/cm^2^ or 0.3 mg/ml solution of collagen in sterile water at 5 µg/cm^2^. The extra solution was aspirated and dishes or plates were dried in UV lamp exposure and washed two times with phosphate‐buffered saline (PBS).

### 2.7 Isolation of mouse primary myoblasts

Mouse primary myoblasts were isolated from 3‐day‐after‐birth mouse hind limb skeletal muscles. Muscles were separated from the bones and fat and subjected treatment with 1.5 U/ml collagenase D and 2.4 U/ml dispase solution (1:1) for 1 hr to isolate myoblasts. Isolated myoblasts were centrifuged at 300 *g* for 5 min and resuspended in growth medium consisting of Ham's F‐10 medium with 10% calf serum (GIBCO), 1% penicillin‐streptomycin, and 2.5 μg/ml fibroblast growth factor. Mouse primary myoblasts were cultured in poly‐L‐ornithine (PLO, Sigma) coated dish for culture, or collagen (Santa Cruz Biotechnology) coated dish to induce myogenesis at 37 ℃ in a humidified 5% CO_2_ atmosphere. When the mouse primary myoblasts reached 90% confluence, the culture medium was replaced with Dulbecco's modified Eagle's medium supplemented with 2% horse serum (Gibco) and 1% streptomycin/penicillin. When inducing myogenesis, the medium was replaced with fresh medium every day.

### 2.8 Immunoblot analysis

After at least 3 days of differentiation, the C2C12 cells or mouse primary myoblasts were lysed with cold tris‐triton lysis buffer. Identical amounts of protein were separated by sodium dodecyl sulfate‐polyacrylamide gel electrophoresis (SDS‐PAGE) and transferred to nitrocellulose membranes. The membranes were blocked in 5% skim milk in PBS with 0.1% tween 20 (PBST) for 1 hr and incubated with primary anti‐Myh3 (1:500, SC‐53091, Santa Cruz), anti‐MyoG (1:500, SC‐52903, Santa Cruz), and anti‐GAPDH (1:2,000, SC‐47724, Santa Cruz) antibodies for 12 hr. After washing the membranes in PBST, immunoreactive signals were obtained using horseradish peroxidase‐conjugated secondary antibodies (Abcam) and Super Signal system (Pierce Chemical).

### 2.9 Immunofluorescence

Mouse primary myoblasts were plated in a 4‐well collagen‐coated chamber slide, and myogenesis was induced for 5 days. The myoblasts were fixed with 4% paraformaldehyde for 10 min, permeabilized with 0.5% Triton X‐100 in PBS for 15 min, blocked with 5% goat serum for 1 hr, and incubated overnight with primary antibodies against Myh3 (1:250, SC‐53091, Santa Cruz) at 4 °C. Alexa Fluor 488‐conjugated goat antibody (1:500, Thermo Fisher Scientific) against mouse IgG was used as the secondary antibody. Nuclei were co‐stained with DAPI (1:1,000; Thermo Fisher Scientific).

### 2.10 Statistical analysis

All results are expressed as the mean ± standard error of the mean of at least three separate experiments for each group. Comparisons between experimental groups were performed using two‐tailed Student's *t* test (SPSS software, version 12.0; BMI). Significance was set at *p* < 0.05.

## 3 RESULTS

### 3.1 Physical characteristics and food supplements

**FIGURE 1:**
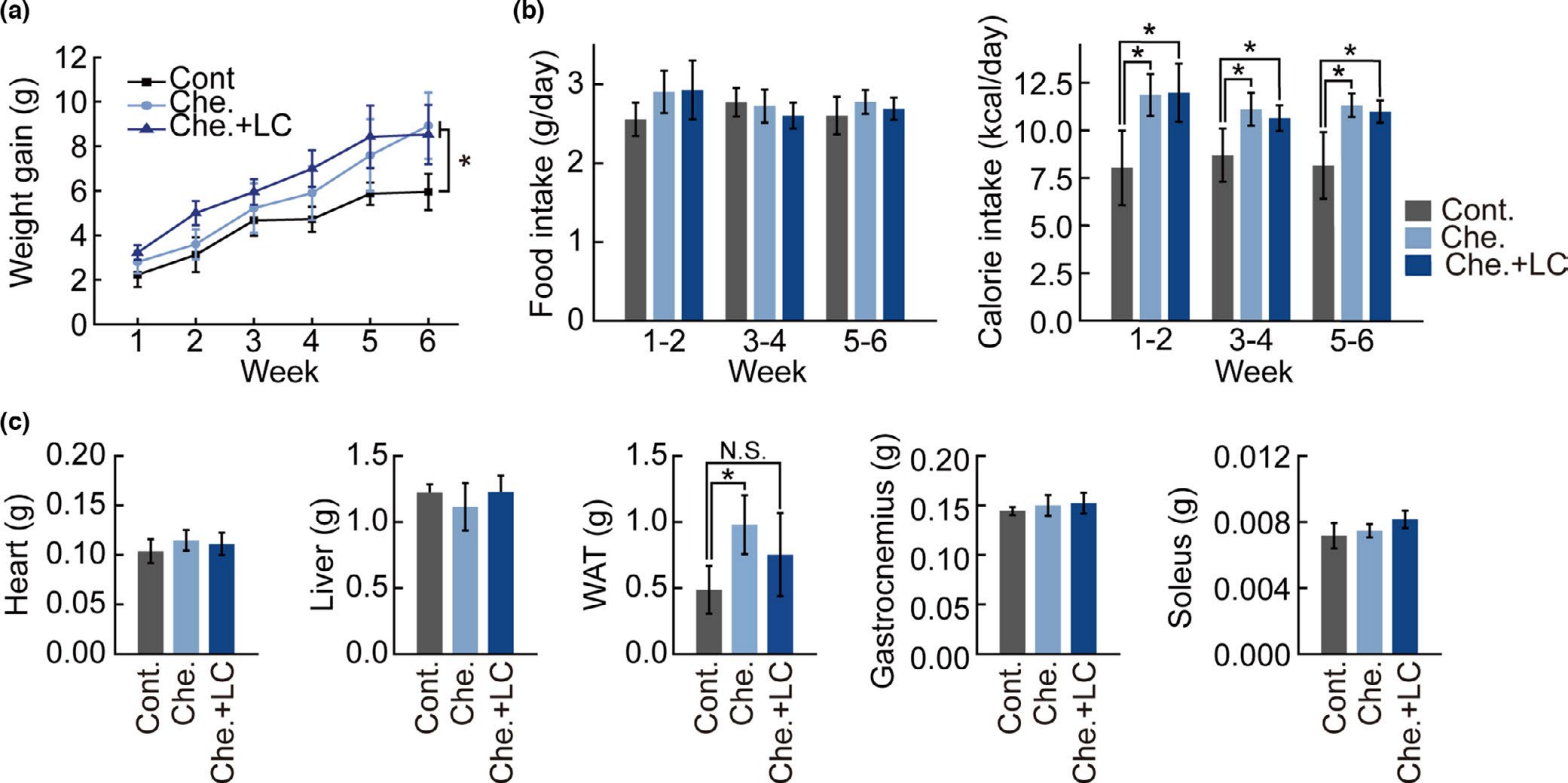
Changes in body weight, food intake, and relative organ weights of mice fed experimental diets. Mice were provided normal diet, cheese (40% of feed), or cheese containing *L*. *chinense* (40% of feed) for 6 weeks. (a) Body weights and weight gain were measured every week. (b) Intake of food and calorie were measured every 3 days. (c) Tissue/organ weights were measured as representative organ. All experimental data are presented as means ± standard deviation (*n* = 4/group). N.S., not significant; *, *p* < 0.05 (paired *t* test)

To compare the protein and fat contents of cheese‐containing diets, we quantified the nutrients in the experimental diets (Table 1). The carbohydrate content of Cont. group (49.1 g/100 g) was higher than that of the Che. (30.5 g/100 g) and Che. + LC (30.4 g/100 g) experimental diet groups. Protein content of the Che. (25.4 g/100 g) and Che. + LC (24.8 g/100 g) experimental diets groups was higher than that of the Cont. group (20.1 g/100 g). Fat content of Che. (20.5 g/100 g) and Che. + LC (20.9 g/100 g) experimental diets groups was higher than that of the Cont. group (4.2 g/100 g). Calorie from the fat of Che. (45.2% of total calories), and Che. + LC (46.0% of total calories) groups was approximately fourfold higher than that of the Cont. (11.9% of total calories) group. As the experimental groups were fed high‐fat diets, we investigated whether experimental high‐fat diets induced weight gain or metabolic change in each mouse group by measuring bodyweight after 6‐week ad libitum feeding. We found that the mouse body weights increased by 8.9 and 8.5 g in the Che. and Che. + LC groups, respectively, whereas it increased by 5.9 g in the Cont. group (Figure 1a). Although the food intake did not differ among the groups, the calorie intake of Che. and Che. + LC groups was higher than that of the Cont. group (Figure 1b). As the calorie intake of the experimental groups was higher than that of the Cont. group, we measured the weight of the heart, liver, FAT, gastrocnemius (GAS), and soleus (SOL) muscles to examine metabolic changes in the three groups. Although the heart, liver, and GAS muscle mass did not differ among the groups, fat mass in the Che. group (0.98 ± 0.22 g) was higher than that in Cont. (0.49 ± 0.18 g) and Che. + LC groups (0.75 ± 0.31 g) (Figure 1c). Moreover, the soleus muscle mass was slightly increased in the Che. + LC group (8.15 ± 0.52 mg) compared to that in the Cont. (7.16 ± 0.76 mg) and Che. groups (7.46 ± 0.39 mg). These results suggest that *L*. *chinense* extracts decreased fat mass and increased SOL muscle mass, whereas the other organs and tissue masses remained unaffected.

**TABLE 1:** Composition of experimental diets

	Cont.	Che.	Che. + LC
Nutrients			
Carbohydrate			
g/100 g	49.1	30.5	30.4
% of total calorie	62.5	29.9	29.8
Protein			
g/100 g	20.1	25.4	24.8
% of total calorie	25.6	24.9	24.3
Fat			
g/100 g	4.2	20.5	20.9
% of total calorie	11.9	45.2	46.0
Total calorie (kcal/100 g)	314.2	408.4	409.3

### 3.2 Serum levels of lipids, GOT, BUN, and blood glucose

**FIGURE 2:**
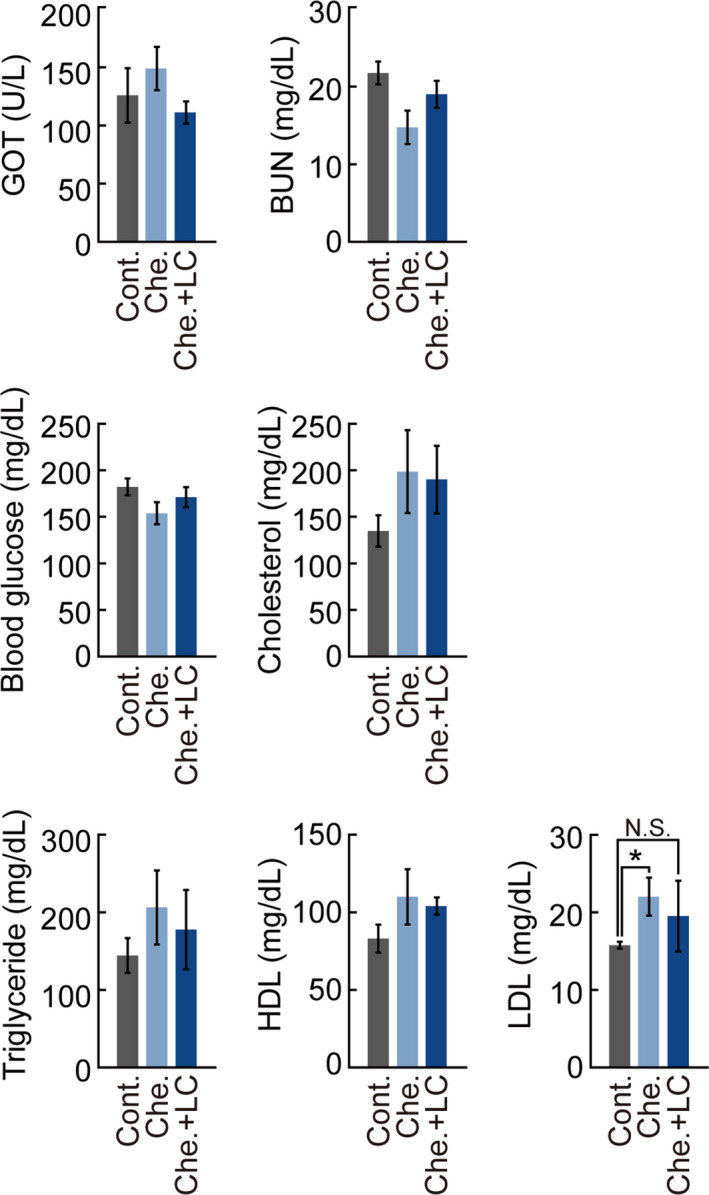
Blood profile of mice fed experimental diets. Changes in GOT, BUN, blood glucose, cholesterol, triglyceride, HDL, and LDL levels were measured. BUN, blood urea nitrogen; GOT, glutamic‐oxaloacetic transaminase; HDL, high‐density lipoprotein; LDL, low‐density lipoprotein. All experimental data are presented as means ± standard deviation (*n* = 4/group). N.S., not significant; *, *p* < 0.05 (paired *t* test)

As the fat mass in the *L*. *chinense*‐fed group decreased and SOL muscle mass increased, we examined the metabolic changes in the groups by serum analysis. GOT, BUN, blood glucose, cholesterol, triglyceride, HDL, and LDL levels were compared among the groups (Figure 2). GOT, BUN, and blood glucose levels were normal and within the clinical reference intervals: 140.6 ± 67.4 U/L for GOT, 22.1 ± 5.3 U/L for BUN, and 206 ± 32 mg/dl for glucose. However, the LDL levels of Che. group (22.0 ± 2.4 mg/dl) was higher than that of the Cont. (15.7 ± 0.4 mg/dl) and Che. + LC groups (19.5.0 ± 4.5 mg/dl). However, the levels of cholesterol, triglyceride, and HDL were similar among the groups. These results show that *L*. *chinense* extracts decrease LDL levels without any significant harmful effects.

### 3.3 Muscle fiber composition and muscle endurance function

**FIGURE 3:**
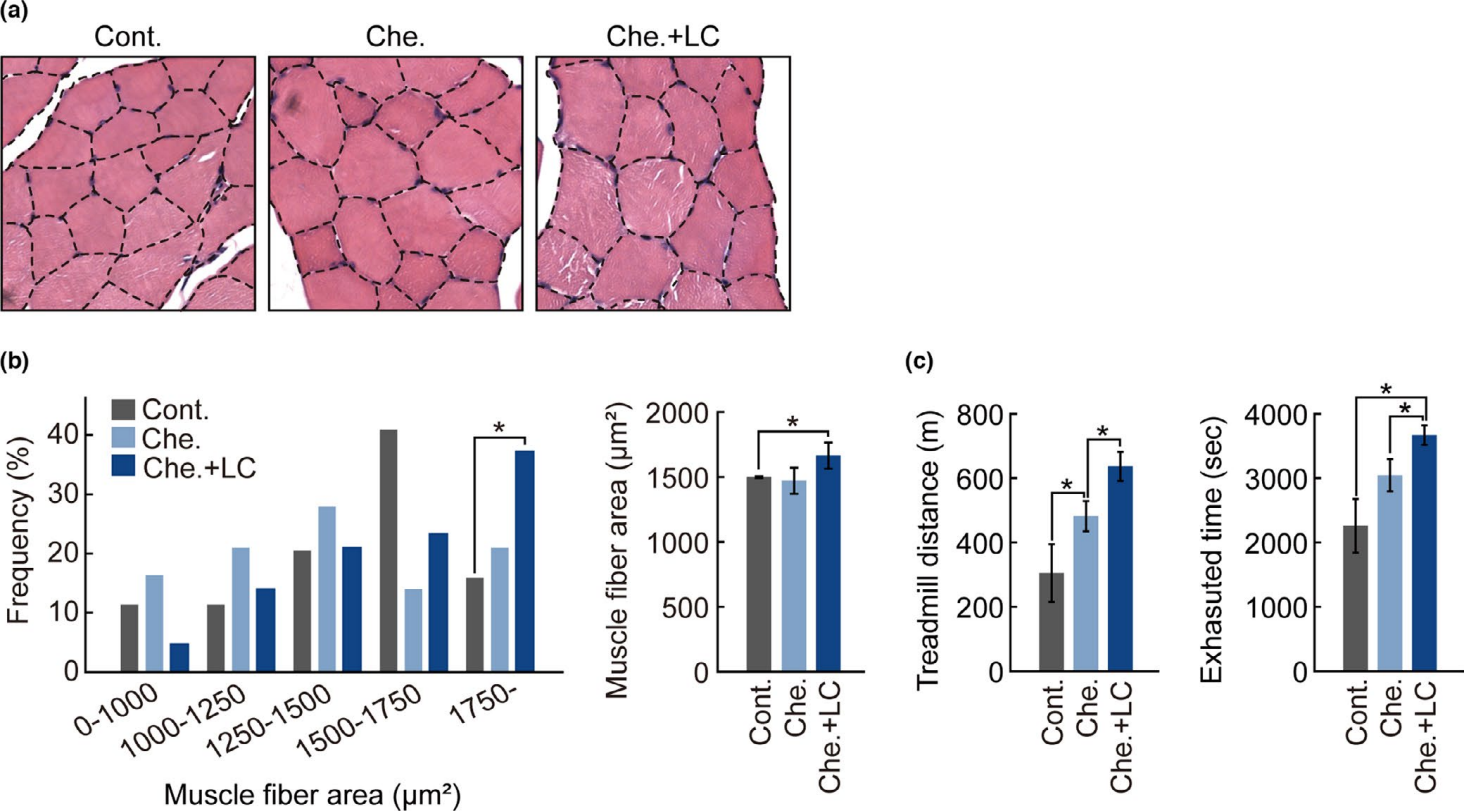
Changes in muscle fiber composition and exercise function in mice fed experimental diets. (a) Representative histological image of hematoxylin and eosin‐stained cross‐section of gastrocnemius muscle in experimental mice. (b) Cross‐sectional area of myofiber in gastrocnemius muscle from each group. Frequency distributions of fibers (left), and measurement and comparison of average fiber area (right). (c) Treadmill endurance tests were performed with progressively increasing speed and time. All experimental data are presented as means ± standard deviation (*n* = 4/group). *, *p* < 0.05 (paired *t* test)

As the SOL muscle mass was increased in *L*. *chinense*‐fed mice (Figure 1c), we measured the distribution of muscle fiber size and thickness of GAS muscles by H&E staining (Figure 3a). The fiber size distribution showed a shift toward larger fiber diameter (Figure 3b). Composition of the hypertrophic muscle fibers (˃1,750 μm^2^) in Che. + LC group (37.5%) was also higher than that of the Cont. (20.9%), and Che. groups (14.3%). Further, the average muscle fiber area of Che. + LC group (1,665.1 ± 141.9 μm^2^) was larger than that of the Cont. (1,478.0 ± 23.9 μm^2^) and Che. groups (1,456.4 ± 105.4 μm^2^). The observation of these histological changes in skeletal muscle in the *L*. *chinense‐*fed mice prompted us to analyze the muscle endurance function via a treadmill test (Figure 3c). We measured running distance and time until exhaustion. The results of running distance before exhaustion showed that the Che. + LC (636.7 ± 45.0 m) group ran more than the Cont. (305.0 ± 90.2 m) and Che. groups (481.7 ± 47.1 m). These results suggest that *L*. *chinense* extract improves the fiber size and endurance function of the GAS muscles.

### 3.4 Activity of betaine on myogenesis

**FIGURE 4:**
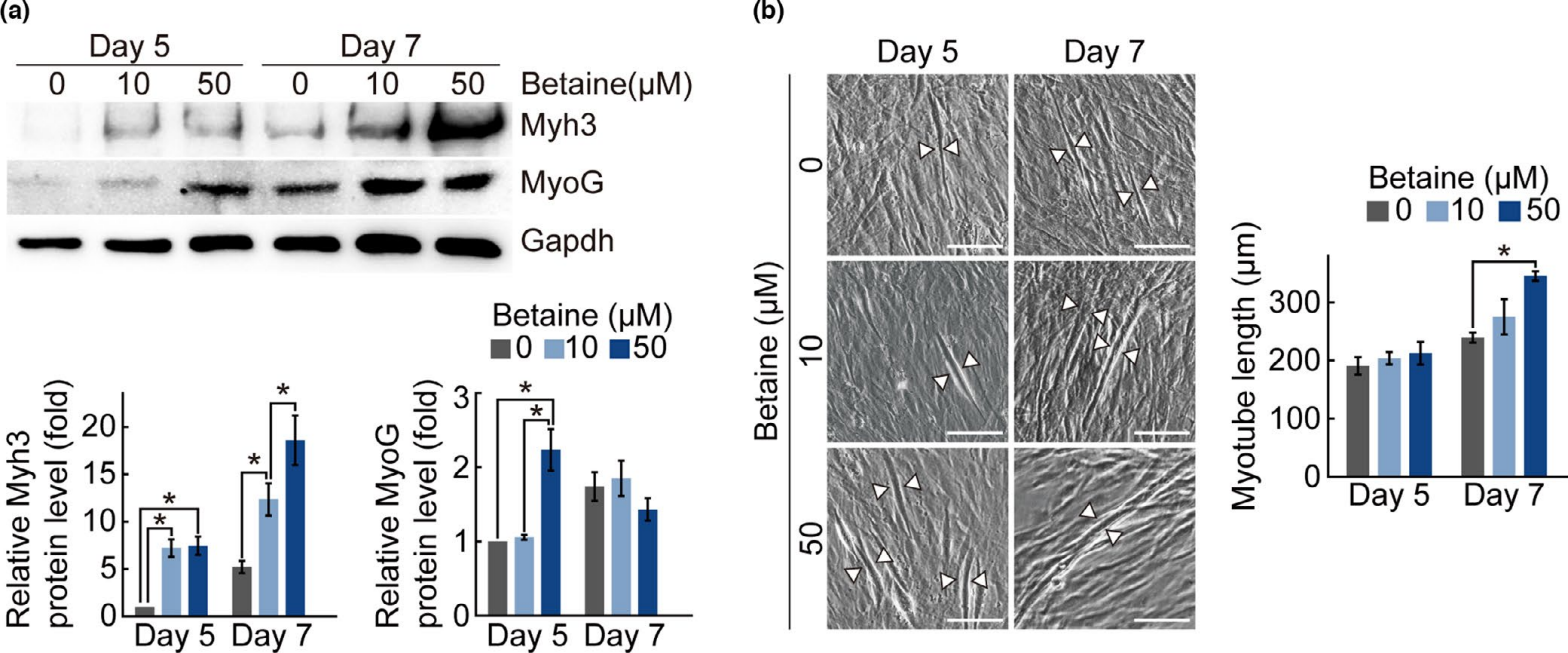
Effect of betaine on myogenesis of C2C12 cells. C2C12 cells were cultured for the indicated periods in differentiation medium containing betaine. (a) Immunoblot analysis was performed with anti‐myosin heavy chain (Myh3) and anti‐myogenin (MyoG) antibodies. Anti‐Gapdh served as an internal control. Relative band intensities of Myh3 and MyoG were quantified using ImageJ software. (b) Representative images of myotubes were obtained at the indicated time points. Length of myotubes from each condition was quantified. All experimental data are presented as means ± standard deviation. *, *p* < 0.05 (paired *t* test)

**FIGURE 5:**
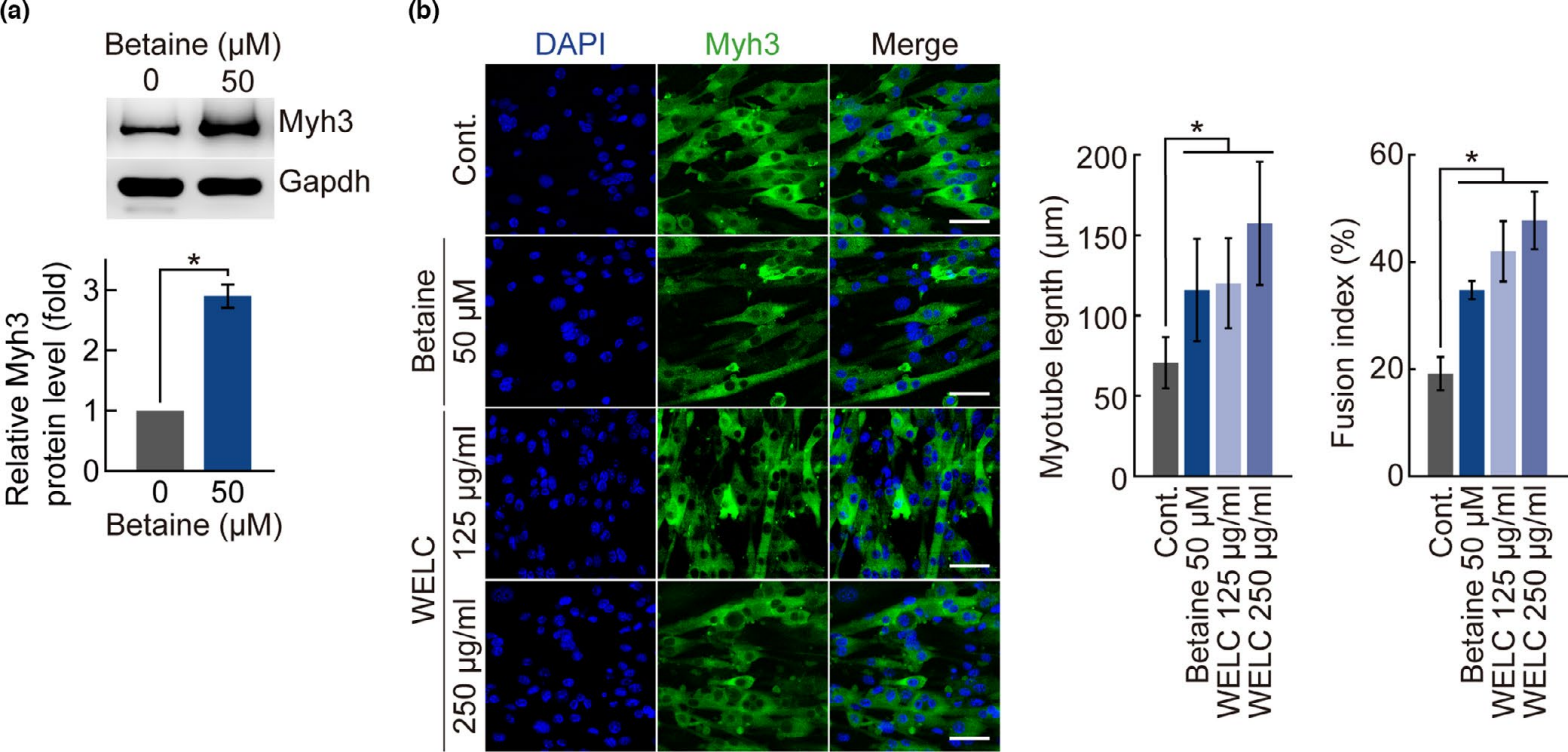
Effect of betaine and *L*. *chinense* on myogenesis of primary mouse myoblasts. Primary mouse myoblasts were differentiated under the indicated condition for 5 days. (a) Immunoblot analysis was performed with anti‐Myh3 antibody. Anti‐Gapdh served as an internal control. Relative band intensities of Myh3 were quantified using ImageJ software. (b) Primary mouse myoblasts were observed by immunofluorescence microscopy. Myh3 (green) and DAPI (blue) were analyzed as representative markers for myotube and nuclear staining, respectively. Length of myotubes and fusion index were quantified. All experimental data are presented as means ± standard deviation. *, *p* < 0.05 (paired *t* test)

Because betaine is a marker of *L*. *chinense* extracts, we investigated whether betaine is a key factor in muscle improvement. We analyzed the betaine content in WELC by HPLC and found that 1 mg of WELC contained 14.72 μg of betaine (Figure S1). Betaine showed no cytotoxicity in C2C12 cells at concentrations of up to 5,000 μM (Figure S2). C2C12 cells were differentiated in the presence of betaine for the indicated number of days, and Myh3 and MyoG protein levels were examined by immunoblot analysis. MyoG expression increased 2.23‐fold on day 5 after treatment with 50 μM betaine (Figure 4a). Myh3 expression was also increased by betaine treatment in a dose‐dependent manner. In addition, betaine treatment increased myotube length in a dose‐dependent manner (Figure 4b). These results suggest that betaine is an effective substance in *L*. *chinense* extracts that is involved in myogenesis. As myogenesis of C2C12 cells was promoted by betaine treatment, we examined the effect of betaine and WELC on myogenesis of mouse primary myoblasts. Mouse primary myoblasts were differentiated in the presence of 50 μM betaine. Myh3 expression was significantly increased by betaine treatment (Figure 5a). In addition, the function of betaine in promoting myotube formation in C2C12 cells was confirmed in mouse primary myoblasts (Figure 5b). Taken together, *L*. *chinense* and its marker betaine enhance myogenesis and muscle endurance in mice.

## 4 DISCUSSION

Skeletal muscle is one of the main organs in the body that performs various functions, such as protecting bones, providing muscle strength, and is important for maintaining health (Danielewicz et al., 2019). Nutrients, including proteins, are among the most important factors in maintaining skeletal muscle (Chanet et al., 2017; Hamarsland et al., 2019). Several recent studies have focused on the effect of continuous intake of foods rich in nutrients, including protein and saturated fatty acids, such as cheese, on improvement in muscle function. *L*. *chinense*, a herb cultivated all over Asia, has been used as a herbal supplement for a long time and contains several physiologically active substances including a high content of betaine, which is well known to have a positive effect on muscle formation. The purpose of this study was to determine the effect of *L*. *chinense* on muscle function under protein‐rich dietary conditions via intake of cheese.

The skeletal muscle is comprised of slow and fast muscles, which affect endurance and quickness, respectively (Sciote et al., 1994). Therefore, to analyze muscle function, appropriate experimental methods need to be selected and analyzed. Methods for evaluating the muscle function of skeletal muscles include a treadmill test or a wheel running test to evaluate muscle endurance, and a grab test to evaluate grip strength (Kim et al., 2020). In this study, we evaluated muscle function through a treadmill test and confirmed that the group that consumed *L*. *chinense* had the most outstanding muscle function. Given that among the various methods of evaluating muscle functions, the treadmill test is particularly suitable for evaluating muscle endurance, it is significant to note that muscle endurance was significantly increased in rats that consumed *L*. *chinense*. Once it was confirmed that muscle endurance, a function of the slow muscles, was improved, the gastrocnemius muscle, which has a high proportion of slow muscle among the leg muscles of the mouse, was analyzed. Although there was no significant difference in its weight compared to that of the other muscles, cross‐sectional analysis revealed that the average thickness of the muscle fibers of the *L*. *chinense‐*fed group increased, and the amount of muscle fibers with a thickness of 1,750 μm^2^ or more was also increased. Since the function of the muscle tissue is strongly influenced by the thickness of each muscle fiber than the amount of muscle fiber, this result shows that the average thickness of the slow muscle fibers of the *L*. *chinense‐*fed group increased, resulting in an increase in muscle endurance. In addition, analysis of the weights of the major organ and various blood indices did not reveal any significant changes, indicating that WELC increased muscle endurance without any toxicity to mouse metabolism.

Myogenesis is a process in which myoblasts form myotube cells through cell fusion and eventually form myofibers. Progression of myogenesis is marked by changes in the expression of myogenic protein and therefore it is possible to evaluate myogenesis based on these changes (Venuti et al., 1995; Whalen et al., 1981). In this study, MyoG and Myh3 were selected as myogenesis indicators. The expression of MyoG and Myh3 were confirmed to be increased in C2C12 cells and primary myoblasts when treated with WELC and its constituent, betaine. In addition to the overall increase in the expression of Myh3, the expression level of MyoG was slightly increased on the 3rd day and decreased again on the 5th day, confirming that myogenesis was significantly promoted. MyoG expression was increased in the early stage of myogenesis and decreased after the myotube was formed (Li, 2014; Yablonka‐Reuveni & Paterson, 2001). Immunofluorescence analysis confirmed that the cells with higher expression of myh3 formed larger myotubes compared to other cells treated with *L*. *chinense* and betaine, and it was confirmed that the number of nuclei contained in each myotube also increased. During myogenesis, myoblasts form long and large myotubes through cell fusion composed of myosin heavy chains. Treatment with *L*. *chinense* extracts and betaine promoted cell fusion and myotube formation in myoblasts, thereby facilitating myogenesis.

Currently, studies on substances that improve sarcopenia and muscle loss are limited. The efficacy of *L*. *chinense* extract and its indicator substance, betaine, in promoting myogenesis, that was confirmed through the results of this study adds significant value by contributing to basic knowledge that may be used for further research on the prevention and treatment of sarcopenia. In addition, considering that *L*. *chinense* is cultivated in a wide area, it can be potentially used to improve sarcopenia more effectively through the development of functional foods that strengthen muscle function.

## CONFLICT OF INTEREST

The authors have declared no conflicts of interest.

## AUTHOR CONTRIBUTION

**Sang‐Soo Lee:** Data curation (equal); Formal analysis (equal); Methodology (equal); Visualization (equal); Writing‐original draft (equal). **Yong‐An Kim:** Data curation (equal); Methodology (equal); Visualization (equal). **Bokkee Eun:** Resources (equal). **Jayeon Yoo:** Resources (equal). **Eun‐mi Kim:** Resources (equal). **Kee K. Kim:** Funding acquisition (equal); Project administration (equal); Supervision (equal). **Myoung Soo Nam:** Resources (equal).

## ETHICAL APPROVAL

Research and animal care protocols were approved by the Animal Experimental Ethics Committee of the Chungnam National University (Daejeon, Korea, approval no. 202009‐CNU‐158) and were performed in accordance with the institutional guidelines.

## Supporting information

Supplementary Material

## Data Availability

The data that support the findings of this study are available on reasonable request from the corresponding author. The data are not publicly available due to privacy or ethical restrictions.
