# Supplementary material for: Betaine, a component of Lycium chinense, enhances muscular endurance of mice and myogenesis of myoblasts
Source: Food Sci Nutr. 2021 Jul 14;9(9):5083–91. doi: 10.1002/fsn3.2466 (PMC8441376; doi:10.1002/fsn3.2466)
Supplement: Supplementary file 1 — Supplementary Material [file FSN3-9-5083-s001.docx]

**Betaine, a component of *Lycium chinense*, enhances**

**muscular endurance of mice and myogenesis of myoblasts**

Sang-Soo Lee^1*^, Yong-An Kim^1*^, Bokkee Eun^2^, Jayeon Yoo^3^, Eun-Mi Kim^4^, Myoung Soo Nam^5#^, Kee K. Kim^1#^,

^1^Department of Biochemistry, Chungnam National University, Daejeon 34134, Republic of Korea

^2^Core Laboratory for Convergent Translational Research, Korea University College of Medicine, Seoul 02841, Republic of Korea

^3^National Institute of Animal Science, RDA, Wanjugun, Jeolabukdo, 55365 Republic of Korea

^4^Department of Predictive Toxicology, Korea Institute of Toxicology, Daejeon, South Korea

^5^Division of Animal Resource Science, Chungnam National University, Daejeon 34134, Republic of Korea

*, #Authors contributed equally.

**Determination of betaine by HPLC.** Betaine contents of WELC was measured via high-performance liquid chromatography (HPLC, Agilent, USA) method with evaporative light scattering detection (ELSD, alltech, USA) [1]. A atrantis silia HILIC 100Å column (150 x 4.6 mm^2^, 5μm) was used. The analysis was performed by ELSD detection. The mobile phase was used as an isocratic elution of acetonitrile and 30 mM of ammonium acetate buffer (pH 3.0 adjusted by acetic acid) (80/20, v/v) until 30 min with 1.0 ml/min..

**Cytotoxicity assay.** To determine cytotoxicity against C2C12 Cells, 3-(4,5-dimethylthiazol-2-yl)-5-(3-carboxymethoxyphenyl)-2-(4-sulfophenyl)-2H-tetrazolium (MTS) assay was performed. C2C12 cells were seeded in 96-well plate. C2C12 cells were maintained for 24 h and treated with sample in concentration gradients for another 24 h. Then, MTS solution (Promega, USA) was treated and absorbance at 490 nm was measured with microplate reader (Molecular Devices EMax Plus, USA).


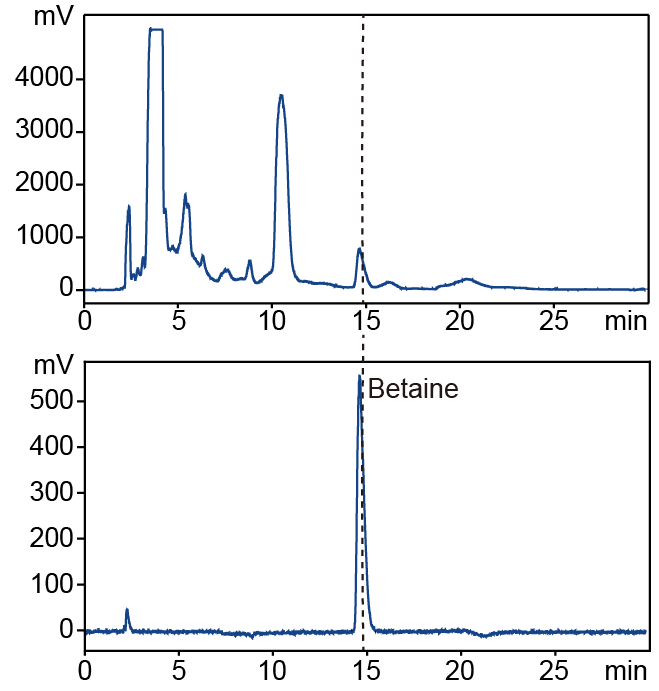


**Supplementary Figure S1.** High-performance liquid chromatography chromatograms of Lyicum chinense extract (upper) and betaine standard (below).


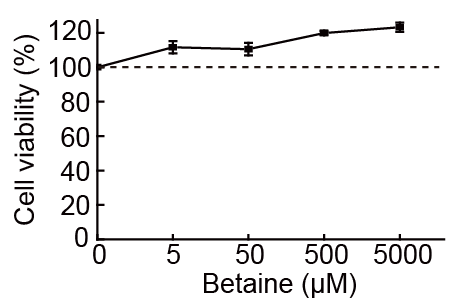


**Supplementary Figure S2.** Cytotoxicity of betaine on C2C12 myoblasts.

**REFERENCE**

1. Zhao, B.T.; Jeong, S.Y.; Hwangbo, K.; Moon, D.C.; Seo, E.K.; Lee, D.; Lee, J.H.; Min, B.S.; Ma, E.S.; Son, J.K.; et al. Quantitative analysis of betaine in Lycii Fructus by HILIC-ELSD. *Arch Pharm Res* **2013**, *36*, 1231-1237, doi:10.1007/s12272-013-0148-9.
